# Anti-oxidant polydatin (piceid) protects against substantia nigral motor degeneration in multiple rodent models of Parkinson’s disease

**DOI:** 10.1186/1750-1326-10-4

**Published:** 2015-03-02

**Authors:** Yupin Chen, Dong-qi Zhang, Zhong Liao, Bin Wang, Suzhen Gong, Chuang Wang, Ming-zi Zhang, Guo-hua Wang, Huaibin Cai, Francesca-Fang Liao, Jiang-ping Xu

**Affiliations:** Department of Pharmacology, School of Pharmaceutical Sciences, Southern Medical University, Guangzhou, 510515 China; Spinal Cord Surgery, Fuzhou Second Hospital Affiliated to Xiaman University, Fuzhou, 35007 China; Department of Pharmacology, University of Tennessee Health Science Center, Memphis, TN 38164 USA; Transgenics Section and Bioinformatics Core, Laboratory of Neurogenetics, National Institute on Aging, Bethesda, MD 20892 USA

**Keywords:** Resveratrol derivative, Piceid, Oxidative stress, Anti-oxidants, Parkinson’s disease, Dopaminergic neurodegeneration, Rotenone, Thioredoxin, MPTP, 6-OHDA

## Abstract

**Background:**

Compelling evidence suggests that inhibition of the complex I of the electron transport chain and elevated oxidative stress are the earliest events during the pathogenesis of Parkinson’s disease (PD). Therefore, anti-oxidants, especially those from natural sources, hold good promise in treating PD as demonstrated mostly by the studies in rodent models.

**Results:**

Herein, we determined if polydatin (piceid), a natural polyphenol, could exert anti-oxidative activity and attenuate dopaminergic neurodegeneration in three commonly used rodent models of PD. Male Sprague Dawley rats given rotenone subcutaneously for 5 weeks developed all the essential features of PD, including a strong increase in catalepsy score and a decrease in motor coordination activity, starting at 4 weeks. Selective increase in oxidative damage was found in the striatal region as compared to the hippocampus and cortex, accompanied by massive degeneration of dopaminergic neurons in the substantia nigra (SNc). Co-administration of piceid orally was able to attenuate rotenone-induced motor defects in a dose dependent manner, with 80 mg/kg dosage showing even better effect than L-levodopa (L-dopa). Piceid treatment significantly prevented the rotenone-induced changes in the levels of glutathione, thioredoxin, ATP, malondialdehyde (MDA) and the manganese superoxide dismutases (SOD) in striatum. Furthermore, piceid treatment rescued rotenone-induced dopaminergic neurodegeneration in the SNc region. Similar protective effect of piceid was also observed in two additional models of PD, MPTP in mice and 6-OHDA in rats, showing corrected motor functions, SOD and MDA activities as well as p-Akt and activated caspase-3 levels.

**Conclusion:**

In three rodent models of PD, piceid preserves and corrects several major anti-oxidant pathways/parameters selectively in the affected SNc region. This implies its potent anti-oxidant activity as one major underscoring mechanism for protecting the vulnerable SNc neurodegeneration in these models. Taken together, these findings strongly suggest a therapeutic potential of piceid in treating PD.

**Electronic supplementary material:**

The online version of this article (doi:10.1186/1750-1326-10-4) contains supplementary material, which is available to authorized users.

## Background

Parkinson’s disease (PD) is a devastating disease and the second most common neurodegenerative disease, afflicting over four million individuals. Pathologically, it is characterized by the loss of dopaminergic neurons in the substantia nigra pars compacta (SNc), which is attributed to both environmental and genetic factors. Mitochondrial impairment, oxidative stress, α-synuclein aggregation and impaired protein degradation have all been implicated in PD pathogenesis. Compelling evidence suggests that inhibition of the complex I of the electron transport chain and elevated oxidative stress (ROS) are the earliest events, followed by modifications of α-synuclein and proteasomal dysfunction, leading ultimately to neurodegeneration [1]. Therefore, anti-oxidants hold promise in treating PD.

**Figure 1:**
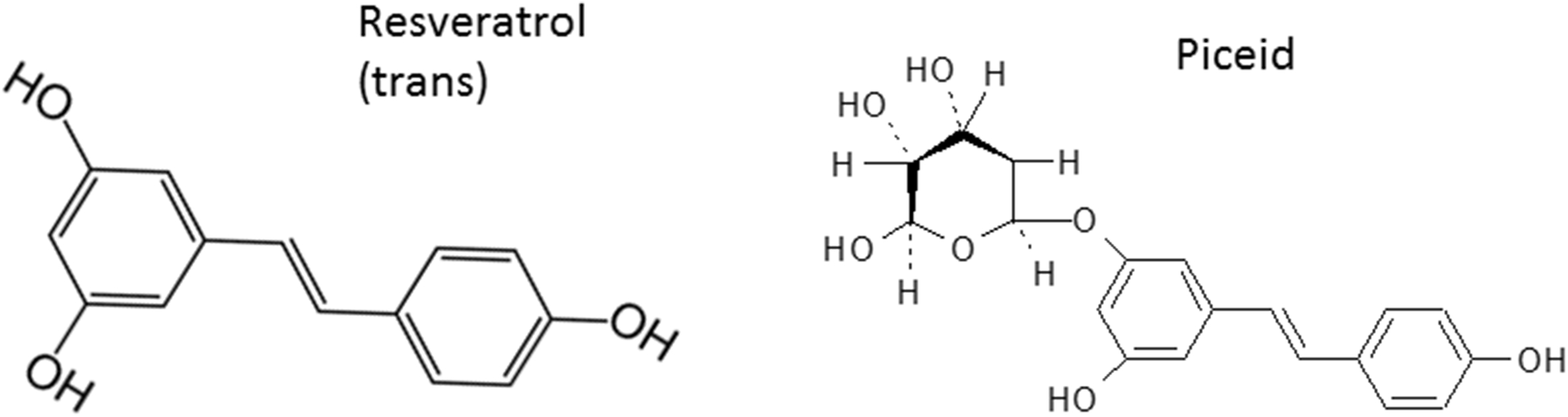
**Chemical structures of trans-resveratrol and piceid.**

It is well-known that a diet rich in fruit and vegetables helps to prevent aging-related disorders, cardiovascular diseases and cancer. This effect may be attributed to the antioxidant activity of polyphenols in fruits and vegetables. Polyphenols, such as resveratrol in grapes, are thought to account for the “French Paradox” which associates red wine consumption with a low incidence of cardiovascular diseases [2]. Despite the identification of multiple beneficial properties (e.g., antioxidant, anti-inflammation, anti-carcinogenic and anti-aging etc.) [3, 4], resveratrol does not display favorable pharmacological properties due to its poor oral bioavailability and rapid clearance rate [5, 6]. Polydatin (piceid, [3, 4′, 5-trihydroxystilbene-3-β-mono-D-glucoside]) is a non-glycosylated derivative of resveratrol [3,4=,5-trihydroxystibene] and is the most abundant form of resveratrol in nature [7] (Figure 1). Piceid’s antioxidant properties are largely unknown. When piceid and resveratrol were compared in terms of their antioxidant properties in lipid peroxidation in one study, piceid appears to be more efficacious [8].

Owing to the fact that it is the most abundant form of resveratrol, it is largely available in non-alcoholic grape juices and is the major extracted component of a widely used traditional Chinese herbal medicine (*Polygonum cuspidatum*), piceid is believed to represent a new medication with modality. The Chinese FDA has approved it for multiple phase II clinical trials in China mainly for anti-shock applications. Piceid has been shown to be neuroprotective in rodent models of focal ischemia [9, 10] as well as in hemorrhage shock treatment [11]. Although resveratrol has been demonstrated to be beneficial in treating stroke [12], spinal cord injury [13] and dopaminergic neurodegeneration in the PD models induced by 6-OHDA and MPTP [14–16], there is no report on piceid in these applications.

In this study, we aimed to evaluate the effect of piceid in treating PD using three rodent models. Rotenone, a commonly used natural pesticide extracted from the roots of tropical plants of the genera *Lonchocarpus* and *Derris*, is a potent specific inhibitor of mitochondrial complex I of the electron transport chain. Chronic systemic administration of rotenone in rodents has recently been demonstrated to be a feasible model of PD by several groups since it induces dopaminergic neurodegeneration, as well as displaying the behavioral, neurochemical (e.g., elevated ROS and depleted ATP in mitochondria) and neuropathological hallmarks of PD [17–22]. However, certain discrepancy has arisen from several recent reports in terms of reproducibility, showing non-selective neurodegeneration or even overall systemic toxicity [23–28]. Therefore, comparing to the MPTP- and 6-OHDA-induced models, rotenone model (especially systemically administrated rotenone) is still not fully validated as a widely used PD model. We therefore focused a great part of our study to develop and validate a systemic rotenone model. After we observed significant protective effect of piceid in MPTP mouse and 6-OHDA rat models, we tested the compound at different dosages and in comparison with L-Dopa (a “gold-standard” treatment of Parkinsonism) in our validated rotenone model; the majority of data are presented from this model.

## Results

### Chronic administered rotenone induced multiple PD-like motor deficits in rats

In a pilot experiment to develop a systemic rotenone model, we chose to test a 2.5 mg/kg/d subcutaneous regimen based on the literature; it appears that most of the studies that generated selective SNc lesion used 2–3 mg/kg/d of rotenone. Subcutaneously (s.c.) injected rotenone at this dosage daily to young adult male SD rats (N = 8) was found to result in severe mortality (~60% mortality) by the end of 4 weeks. We therefore adjusted the dosage to 2.0 mg/kg/d which was found to minimize mortality rate to < 10%; one out of 12 rats was dead. Tolerable weight loss (~20%) was observed during the 4-5^th^ week period. Pilot behavioral tests for motor functions indicated severe impairment induced by this regimen of rotenone; rotenone administration for longer than 5 weeks led to increased mortality and > 25% weight loss Based on these pilot studies, we therefore decided to use 2.0 mg/kg/d rotenone s. c. for 5 weeks in the following experiments.

### Effect of piceid on rotenone-induced catalepsy and motor coordination disorder

**Figure 2:**
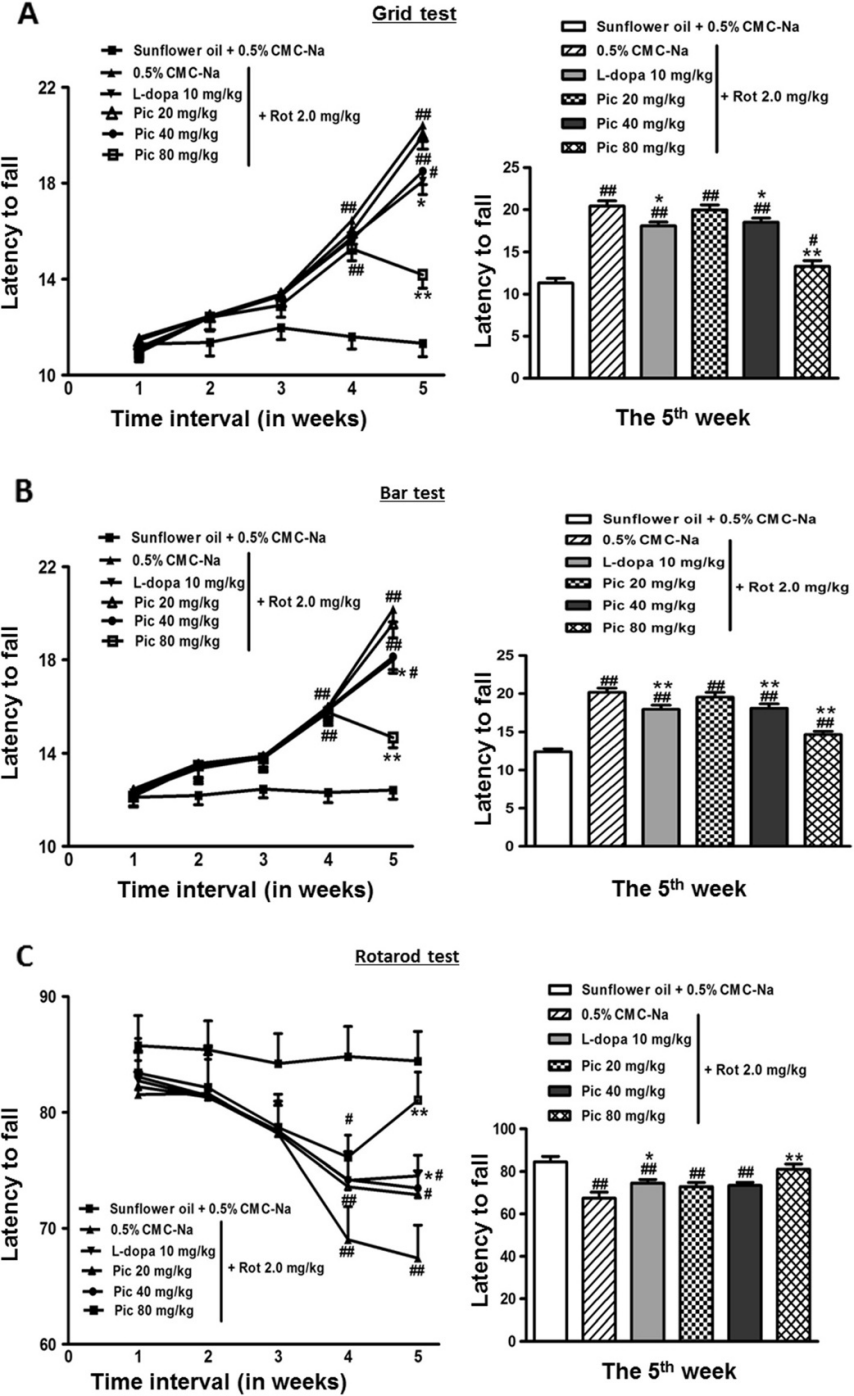
**Effects of piceid and L-dopa on rotenone-induced catalepsy (A: grid test; B: bar test) and motor coordination disorder (C) for rats in their 1st, 2nd, 3rd, 4th, and 5th week of treatment.** Data is mean ± SEM of ten animals. #*p* < 0.05, ##*p* < 0.01 significant as compared to vehicle treated animals of the respective week; *p < 0.05, **p < 0.01, significant as compared to the rotenone treated animals of the respective week. Comparison between the 80 mg/kg/d piceid group and the 10 mg/kg L-dopa group yields *P* < 0.05 for the grid and motor coordination tests and *P* <0.01 for the bar test. Rot: rotenone; Pic: piceid.

During the 5-week acquisition trials in the catalepsy test and rotarod motor coordination test, all the rats given rotenone displayed progressive increases in cataleptic behavior (a measure for rigidity) and decreases in motor coordination and muscle activity as compared to the vehicle group (Figure 2A and B). Prominent progression was observed during the last two weeks, especially in the 5th week (*p* < 0.01). The motor coordination and muscle performance of rotenone-treated rats decreased significantly (Figure 2C).

In our pilot experiments, we tested piceid administration to rats via oral gavage daily in escalating dosages (50–200 mg/kg/d) and found that it was well-tolerated at the high dosage of 200 mg/kg/d after continuous administration for 14 days. Biochemical analysis indicated significantly increased anti-oxidant profiles (Trx, SOD etc.) in all the three regions (hippocampi, cortex and striatum) at these dosages with maximum effect observed at 100 or 200 mg/kg dosages of piceid (data not shown). Like resveratrol [29], piceid is predicted to be able to pass blood–brain-barrier. Indeed, our pilot tissue distribution study supported its CNS permeability, though its distributions in brain and skeletal muscle are much less than that in liver, lung, kidney and intestines (data not shown). Based on these data, we therefore chose a range of dosages between 20–100 mg/kg in our next experiments to be co-administrated with rotenone. Notably, piceid prevented the progressive increases in catalepsy when compared to the untreated rotenone-induced animals in a dose- and time-dependent manner (Figure 2A and B). Based on the rats’ performance at the 5 week time point, the 80 mg/kg piceid group displayed marked reduction (*P* < 0.01) while the 40 mg/kg piceid group showed performance comparable to the 10 mg/kg L-dopa group. For all three tests (Figure 2), 80 mg/kg piceid group performed significantly better than the 10 mg/kg L-dopa group (please see Figure legend for *P* values).

### Effect of piceid on restoring rotenone-decreased ATP

**Figure 3:**
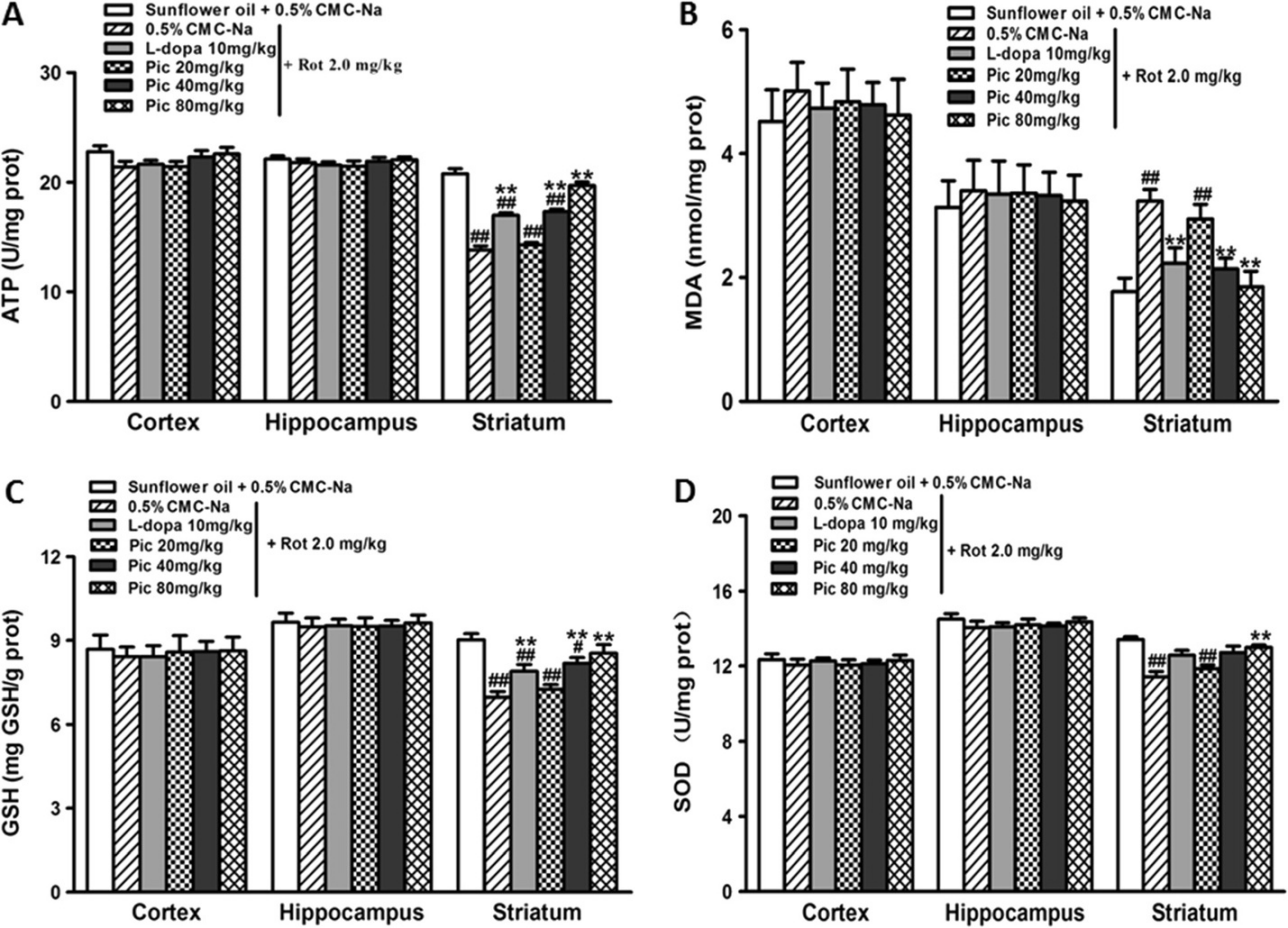
**Effects of piceid and L-dopa on rotenone-induced alterations to the levels of ATP (A), MDA (B), GSH (C), and SOD (D) in different brain regions (Cortex, Hippocampus, Striatum).** Data are expressed as mean ± SEM (n = 9/group). For statistical significance, # and ## indicate *p* < 0.05 and 0.005 as compared to the vehicle control group; ** indicates *p* < 0.005 as compared to the rotenone model group. Significant difference between the 80 mg/kg piceid and 10 mg/kg L-dopa groups is *P* < 0.01.

Rotenone is believed to act on the mitochondrial electron transport chain and selectively inhibits complex I, resulting in a dwindling supply of cellular energy, which has been implicated in the pathogenesis of PD. It was also reported that rotenone completely prevented the oxidation of pyruvate and many other physiological substrates, thereby inhibiting ATP synthesis in mitochondria [30]. We therefore determined the absolute levels of intracellular ATP in the different brain regions (Cortex, Hippocampus, Striatum). As shown in Figure 3A, rotenone selectively reduced intracellular ATP in the striatum by over 40% while high dosages (80 mg/kg) of piceid largely restored the ATP level. These results indicate the potent beneficial effect of piceid in restoring mitochondrial energy levels impaired by rotenone.

### Selective anti-oxidative effect of piceid in striatum

Lipid peroxidation was determined by measuring an end product, MDA, in the different brain tissues after behavioral testing. We found that rotenone increased MDA levels in the striatum by almost 2-fold (*P* < 0.01) but not in the other two brain regions (cortex and hippocampi) (Figure 3B). Treatment with piceid or L-dopa both reduced MDA levels in a dose-dependent manner, with the high dosage (80 mg/kg) of piceid completely restoring it to the basal level (*P* < 0.01). In addition to MDA, we also investigated the other two most important antioxidant defense molecules, GSH and SOD, and found similar results (Figure 3C and D): piceid treatment at 80 mg/kg completely restored the levels of these two molecules impaired by rotenone. Taken together, these findings indicate the selective vulnerability of the striatum to elevated oxidative stress and show the potent effect of piceid in antagonizing ROS.

### Effect of piceid on thiol antioxidant system-thioredoxin (Trx)

**Figure 4:**
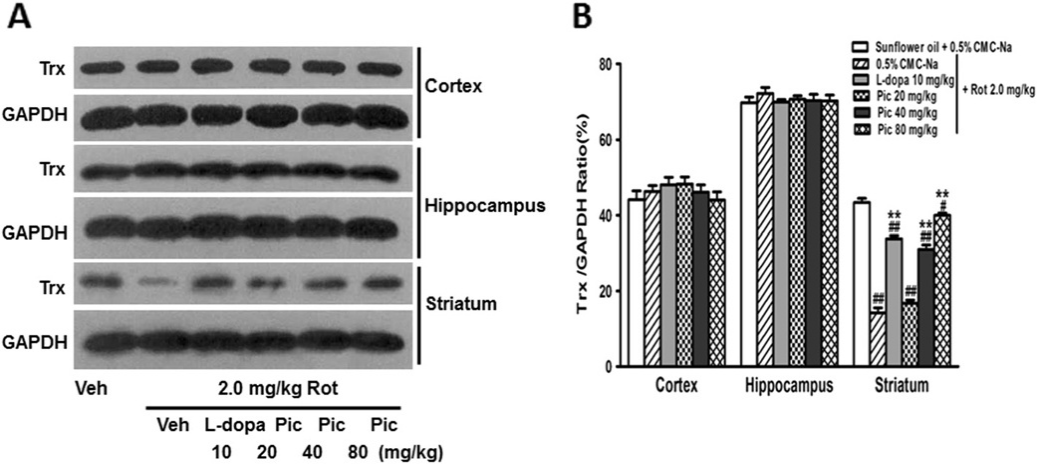
**Effects of piceid and L-dopa on the rotenone-induced alteration of Trx expression in different brain regions (Cortex, Hippocampus, Striatum). A)** Western blot analysis showing representative samples from each group (1 of 9 samples/group). **B)** Quantification based on all 9 samples in each group. Data are expressed as mean ± SEM (n = 9/group). For statistical significance, # and ## indicate *p* < 0.05 and 0.005 as compared to the vehicle control group; ** indicates *p* < 0.005 as compared to the rotenone model group. Significant difference between the 80 mg/kg piceid and 10 mg/kg L-dopa groups is *P* < 0.05.

The Trx system is ubiquitous in all cells and is involved in many redox-dependent signaling pathways [31]. Consistent with the aforementioned ROS parameters, we found selective reduction of the classical 12 kDa Trx protein in the striatum after rotenone induction. This was restored by piceid and L-dopa treatment with the maximum effect achieved by the high dosage of piceid (Figure 4).

### Piceid’s protective effect on SNc neurodegeneration induced by rotenone

**Figure 5:**
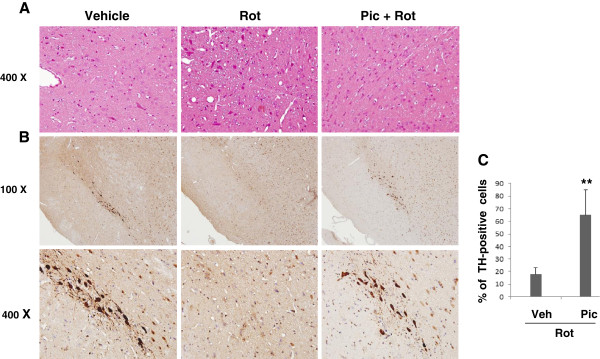
**Selective SNc neurodegeneration induced by rotenone and the rescuing effect of piceid. A)** Coronal sections from the rostral to caudal dopaminergic area containing neurons in the SNc were depicted by H&E staining. **B)** Representative TH-immunostained from coronal sections containing the SNc regions. **C)** Quantification of the TH-immunoreactive neurons based on the scans of every 10^th^ section of the striatum (5 sections/animal × 5 animals per group) using Scion Image (Version 4.0.3.2 Scion Corporation, MD). Vehicle: sunflower oil + 0.5% CMC-Na 10; Pic: piceid (80 mg/kg/d); Rot: rotenone (2.0 mg/kg/d). “**” indicates *P* < 0.01 as compared to the piceid treatment group.

Our data thus far collected from motor behavioral tests and biochemical analysis all suggested selective neuroprotection of piceid in the striatal region. To further validate our systemic rotenone model in generating selective SNc neurodegeneration, we examined the rat tissues collected by the end of our behavioral studies by histological means and found that rotenone selectively induced damage in the striatal region, as evidenced by increased eosinophilic cells and swollen soma revolved by H & E staining (Figure 5A). In contrast, the adjacent regions (cortex and hippocampi) were largely unaffected (data not shown). Rotenone also induced selective and massive loss of the TH-immunostained neurons from the SNc region after 5-weeks (Figure 5B) which was largely rescued by piceid treatment (80 mg/kg) (Figure 5C, *p* < 0.005).

### Piceid also displayed potent anti-oxidant activity and prevented/corrected motor dysfunctions in two additional PD models

**Figure 6:**
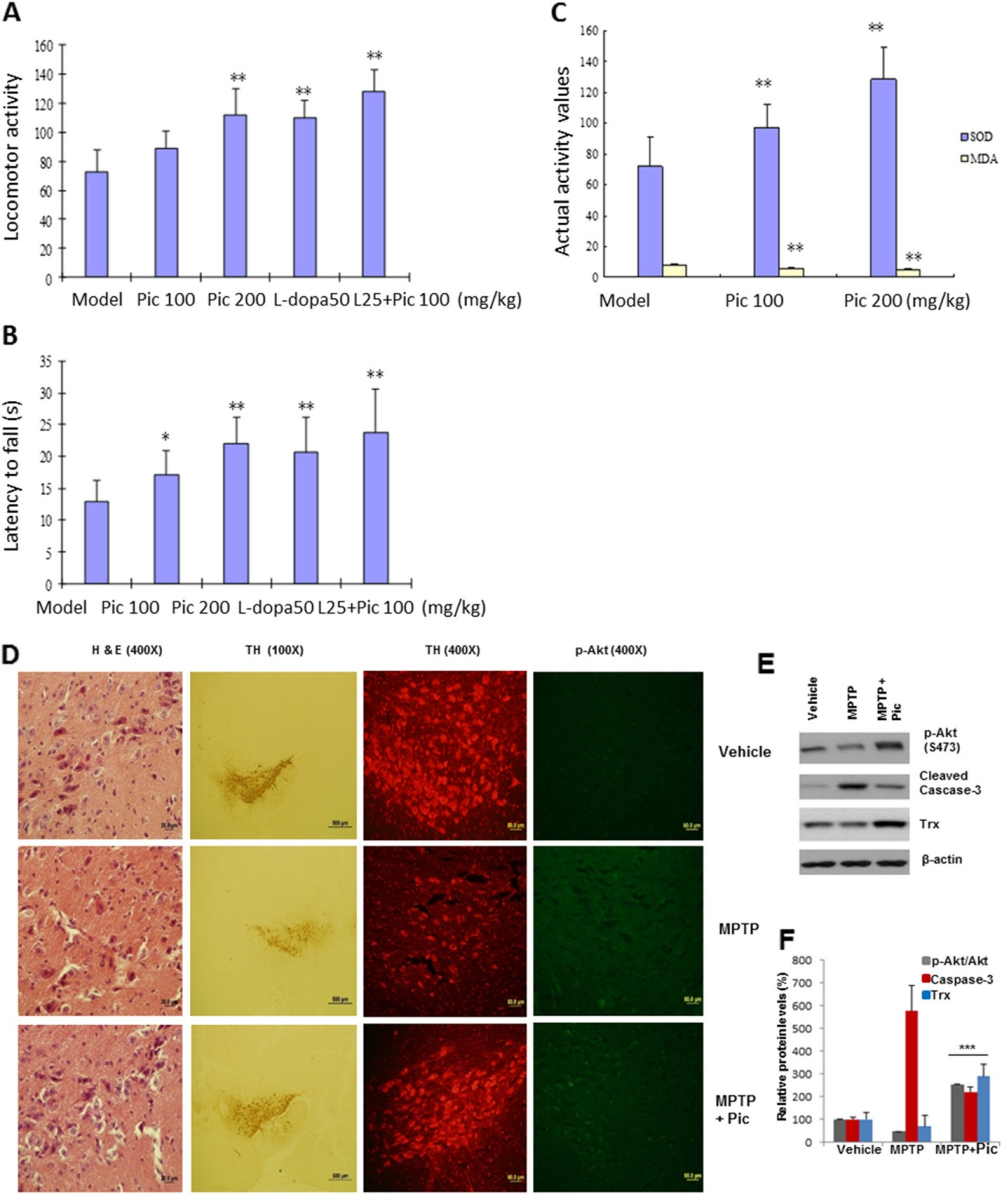
**Effects of piceid and L-dopa on MPTP-induced PD in mice.** Locomotor activity **(A)** and rotarod activity **(B)** on treated and untreated C57BL/6 mice determined on the 15^th^ day (N = 10 animals each troup). **C)** The actual SOD activity and MDA levels. “* and **” indicate *P* < 0.05 and 0.01, respectively, as compared to the MPTP-induced model group. L50 and L25 indicate L-Dopa at 50 and 25 mg/kg dosages, respectively. **D)** Immunohistochemistry. Coronal sections of paraffin blots (6 μcrom) were stained with H & E, mouse anti-TH (both DAB and fluorescent readouts) and antibodies against p-Akt (Ser374) and activated caspase-3. Representative images were chosen from a set of three mice each group. **E)** Western blot analysis of the isolated SNc tissue (lysates pooled from 3 mice each group). Trx: thioredoxin; **F)** Quantification. “***” indicates *P* < 0.005 as compared to the MPTP group.

**Figure 7:**
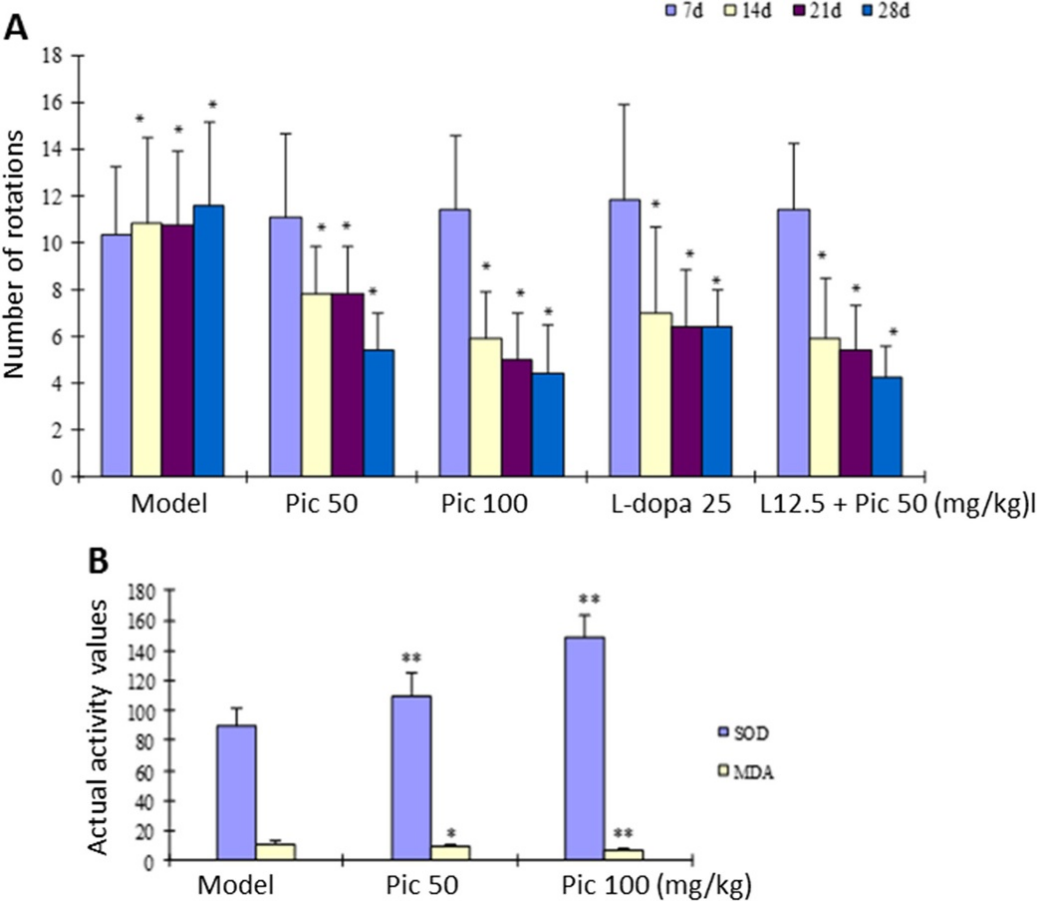
**Effects of piceid and L-dopa on 6-OHDA-induced PD in rats. A)** Number of apomorphine-induced rotations as determined on 7, 14, 21, and 28 days after 6-OHDA injection. N = at least 9 animals per group. * indicates P < 0.05 as compared to the 6-OHDA-induced model group. **B)** The actual SOD activity and MDA levels. * and ** indicate *P* < 0.05 and 0.01, respectively, as compared to the model group.

To verify the usefulness of oral piceid in preventing and treating PD, we also tested the compound in MPTP-induced mouse and 6-OHDA-induced rat models. Daily oral piceid treatment on young male C57BL/6 mice starting from the first day of subcutaneous injection of MPTP (40 mg/kg for 7 days) for 14 days significantly prevented motor deficits and preserved SOD activity (and reduced MDA values) in the striatal region (Figure 6A-C; Additional file 1: Figure S1-S4); 200 mg/kg of piceid performed comparably with 25 mg/kg L-dopa. Interestingly, combination of 100 mg/kg piceid with 12.5 mg/kg achieved the same effect with either compound alone at twice the dosage, indicating that piceid treatment can achieve the same outcome as L-dopa. Furthermore, histological and biochemical analysis of the striatal region indicates markedly protective effect of piceid on TH-positive DA neurons, survival signaling (e.g., increased p-Akt and reduced activated caspase 3, Figure 6D-F). In a 6-OHDA-induced PD model in rats, the apomorphine-induced rotations of the 6-OHDA-lesioned rats were significantly increased (10.89 ± 2.47 vs 0.44 ± 0.73 times/min, n = 10 animals each group; *P* < 0.005). SOD activity (166.51 ± 17.29 vs 89.28 ± 12.79) and MDA levels (5.94 ± 1.65 vs 10.88 ± 2.37) were also dramatically impaired by 6-OHDA (*P* <0.001). Strikingly, significant and sustained effect was achieved with 50 mg/kg/d piceid starting a few hours after 6-OHDA injection from 7 days and up to 28 days (Figure 7A); the effect is comparable (slightly better but no significant) with the 25 mg/kg L-dopa group or 50 mg/kg piceid + 12.5 mg/kg L-dopa. Similarly, the SOD and MDA activity/levels were significantly improved by piceid treatment.

## Discussion

Current treatments for PD are limited in effectiveness and only improve the symptoms of the disease and don’t address the cause. L-dopa, which increases brain levels of dopamine, has been used for years and is the gold standard for treating PD. It is currently the most effective drug for controlling symptoms and is used in nearly all phases of the disease. However, this drug increases the risk of involuntary movements (dyskinesia). Moreover, its effectiveness tends to decrease after 4–5 years of usage [32], making discovery of a new drug imperative [33]. Elevated oxidative stress has been reported in the brains of PD patients [34]. Therefore, combining an antioxidant with other protective agents would be a good therapeutic option for neurodegenerative diseases like PD. Polyphenols represent one of the most promising categories of therapeutic candidates, owing to their potent protective and anti-oxidative properties. Our present work demonstrates that piceid displays strong anti-oxidative property and systemic piceid administration can achieve preventive effects, comparable or better than L-dopa in rescuing PD-like motor deficits induced by rotenone, MPTP or 6-OHDA.

We should emphasize that in all three models, piceid treatment can achieve at least the same beneficial outcomes as with L-dopa in terms of motor behaviors and DA neuronal cells. Although L-dopa is a typical antiparkinsonism acting by increasing dopamine activity, it is not clear whether piceid other than its primary identity as an anti-oxidant, can also exert effect on dopamine release/activity. Our data support its primary role in anti-oxidation and preserving DA neurons. Therefore, the effect of piceid is more likely to be primarily neuroprotective.

### Rotenone-induced PD models in SD rats

Chronic systemic exposure to rotenone, a specific complex I inhibitor and common herbicide, has been shown to reproduce Parkinsonian features in rats. Several research groups have independently demonstrated that systemically administered rotenone causes highly selective nigrostriatal dopaminergic degeneration, which is associated with the neurochemical, behavioral and neuropathological features of PD [17–21]. However, considerable controversy has been reported in terms of the selectivity, overall degree and reproducibility of the dopaminergic degeneration [22–28].

Here, we demonstrated that a sublethal dose of 2.0 mg/kg/d s. c. rotenone for 5 weeks induces the full reproduction of the PD phenotypes, including not only progressive increases in cataleptic behavior and decreases in motor coordination and muscle activity, but also mitochondrial dysfunction and increased ROS and SNc neurodegeneration of the TH-cells selective to the striatal region. In our model, bradykinesia and rigidity are manifested as an increase in the catalepsy score while mitochondrial dysfunction and increased ROS were evidenced by decreased ATP production and significant alteration of several major indices of oxidative stress, including MDA, SOD, GSH and Trx. It should be stressed that all of these neurochemical changes are restricted to the striatal region, which supports the notion that the SNc is more (and probably most) vulnerable to the impairment induced by rotenone, likely owing to impaired complex 1 activity and the subsequently elevated ROS. Finally, severe neuropathological changes were demonstrated in our model by histology showing massive and selective loss of TH-positive dopaminergic (DA) neurons in SNc region. The selective vulnerability of the SNc and DA neurons to rotenone is not fully understood. It is generally believed to be largely due to the increased levels of ROS generated within DA neurons as a result of dopamine metabolism, auto-oxidation and increased iron content [35]. The recent findings of decreased mitochondrial mass in the SNc dopamine neurons, as compared to the mid brain region and frontal cortex in mice [36], may also contribute to the extraordinary vulnerability of the SNc DA neurons, though it is not clear whether this is the case in human brains.

### Potent anti-oxidative effect is likely piceid’s key protective mechanism

Since the original report [37], we have confirmed that L-dopa administration can prevent rotenone-induced severe motor deficits to some degree, based on three independent tests (Figure 2). A comparable and significant beneficial effect was observed with piceid treatment at the 40 mg/kg/d dosage. Notably, piceid treatment at a high oral dosage of 80 mg/kg/d results in a better outcome in alleviating these motor deficits than L-dopa, with a 30% and 27% reduction in the grid and bar tests and > 20% increase in the motor coordination test (Figure 1, 5 week end-point). This is the first report that demonstrates the effect of an abundant polyphenol from natural sources in an animal model of PD.

In Parkinson’s disease, oxidative stress and mitochondrial dysfunction underlie the development of neuropathology. In addition to rescuing rats from rotenone-induced motor behavioral changes, piceid treatment also preserved mitochondrial ATP levels and counteracted rotenone-induced elevated ROS selectively in the striatal region. It is unclear how orally administered piceid can result in a selective effect in the striatal region that presumably correlates with the most rotenone impaired SNc DA neurons. We observed a significant reduction in the levels of an oxidative marker, the lipid peroxidation product MDA, after piceid treatment. Piceid may detoxify peroxidized lipid membranes by directly interacting with the peroxidated lipids to restore them to lipid alcohols. Piceid treatment also significantly attenuated rotenone-induced depletion of GSH in the striatum. A defect in one or more of the naturally occurring antioxidant defenses, particularly glutathione GSH, is an important factor in the etiology of PD [38]. In fact, depletion in the level of the thiol reducing agent GSH is the earliest reported biochemical event to occur in Parkinsonian SNc prior to the selective loss of complex I activity [39]. Depletion of GSH has been consistently observed in the nigra of PD patients who have been diagnosed with incidental Lewy bodies, which has been established as a preclinical symptom of PD [34]. A reduction in GSH levels can further impair H_2_O_2_ clearance and promote hydroxyl radical formation, leading to the generation of a pro-oxidant milieu. Similar to GSH, SOD activity has also been shown to be up-regulated by piceid treatment. Paradoxically, one earlier study has reported an elevation in SOD activity following rotenone exposure [40]. However, in the present study, SOD activity was found to decrease significantly in the striatum following chronic exposure to rotenone. Thus, any of these defects in enzymatic defense systems could lead to an imbalance in antioxidant and oxidant species, resulting in elevated oxidative stress. It would appear that piceid can enhance the decomposition of H_2_O_2_ and dismutation of superoxide anions by increasing the availability/activity of GSH and SOD, thereby reducing the damage likely to result from these devastating free radicals.

The thioredoxin (Trx) system is another antioxidant system we looked at, in addition to the enzymatic defense systems. Thioredoxin reductase (TrxR), in conjunction with Trx, is a ubiquitous oxidoreductase system with antioxidant and redox regulatory roles. Trx, a small protein with two redox-active disulfide/dithiol sites in its conserved active site sequence, −Cys-Gly-Pro-Cys-, has a neuroprotective effect in many experimental systems [31, 41]. There are several reports showing that the beneficial effects of many anti-oxidants depend on their ability to upregulate and activate the Trx system. Consistently, thioredoxin reductase deficiency was found to potentiate ROS and mitochondrial dysfunction and cell death in DA neurons [42]. However, this has not been seen in PD patients’ brains, though Trx is reportedly reduced in PD striatum. Nevertheless, it is tempting to speculate that Trx plays a crucial role in PD. Co-treatment with a high dose of piceid (80 mg/kg) resulted in up-regulation of the Trx protein level in the striatum which completely restored the reduced Trx protein levels induced by rotenone.

**Figure 8:**
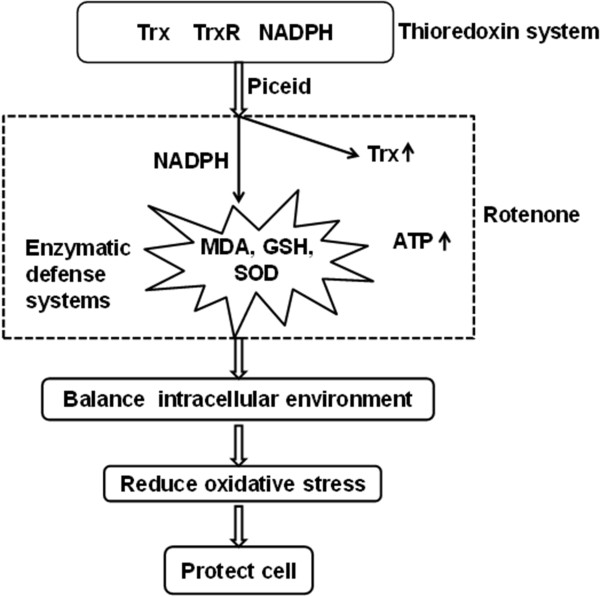
**Diagram elucidating the proposed model of imbalanced ROS initiated by disrupted Trx system.** Please see text for detailed discussion.

It has long been speculated that piceid can reduce oxidative stress like resveratrol. However, as far as we know, there was no detailed mechanism to account for how piceid reduces oxidative stress. Mechanistically, rotenone-induced dopaminergic neurodegeneration has been generally attributed to its inhibition of neuronal mitochondrial complex I [23]. While it is well established that the activity of the mitochondrial respiratory complex I is reduced in the SNc of sporadic PD patients [43], several pieces of recently gathered evidence suggest that rotenone’s effect may not be solely attributed to impaired complex I activity [44–47]. In the SY5Y cellular model, we demonstrated that piceid is a much superior anti-oxidant over resveratrol against hydrogen peroxide (Liao, unpublished data). However, we do not know whether these impairments are secondary, resulting from impaired complex I, nor do we know which system plays the primary role in mediating piceid’s anti-oxidative effect. Based on the increasing knowledge in literatures, it is plausible that Trx system plays a primary role to attenuate the rotenone-induced intracellular redox imbalance, oxidative stress, and eventual cell death as illustrated in Figure 8. Piceid increases the levels of Trx protein, altering the transfer of electrons from NADPH to oxygen and the production of ROS. Furthermore, the lipid peroxidation level is drastically down-regulated after co-treatment with piceid and both the availability of GSH and the activity of SOD are significantly upregulated. Alternatively, it is also possible that the increase in the availability of GSH, the activity of SOD and the up-regulation of Trx protein levels act in combination to mediate the key mechanisms through which piceid exerts its antioxidant effect.

## Conclusions

In summary, the present study demonstrates that chronic administration of rotenone to SD rats produces selective damage to the striatum, possibly by elevating free radical species and impairs the antioxidant system. Piceid co-treatment successfully attenuated most of these changes. We present both *in vivo* and *in vitro* evidence to support that piceid is a strong anti-oxidant in DA neurons. Our work elucidates the tight correlation between the potent anti-oxidative activity of piceid and its protective effect on motor functions. This finding further validates the important role of mitochondrial dysfunction in the pathogenesis of PD in this rodent model, as evidenced by a decrease in striatum ATP levels, which are considered potential biomarkers for the diagnosis of PD [48]. Our results strongly support the possible therapeutic potential of piceid as an antioxidant in PD and other movement disorders where oxidative stress is a key player in the progression of the disease. Our findings add to a growing body of evidence supporting naturally derived anti-oxidants as a new class of therapeutic options [49].

## Methods

### Animals, chemicals and drug treatment

Male Sprague Dawley (SD) rats and C57BL/6 mice of 7–8 weeks were obtained from the Laboratory Animal Center of the Southern Medical University (Certificate No. 20060015; conferred by the Guangdong Medical Laboratory Animal Administration Committee) and were acclimated to the facility for 1 week before the experiments. Animals were housed 6–8 per cage in a temperature controlled room (21 ± 2°C) with a 12-h on /12-h off light cycle, and were given *ad libitum* access to water and food. All procedures were performed in accordance with the US NIH’s “Guide for the Care and Use of Laboratory Animals”, with the approval from the Ethics Committee at the Southern Medical University, Guangzhou, China.

Piceid was kindly provided by Shenzhen Neptunus Pharmaceutical Co., Ltd., China (Cat# N03030301). Rotenone, levodopa (L-dopa), MPTP (1-methyl-4-phenyl-1,2,3,6-tetrahydropyridine), 6-OHDA (6-hydroxydopamine) were purchased from Sigma (St. Louis, MO). Assay kits for determination of malondialdehyde MDA), glutathione (GSH), superoxide dismutase (SOD), and Adenosine triphosphate (ATP) were supplied by Nanjing Jiancheng Biotechnology Institute (Nanjing, China). Rotenone was emulsified in sunflower oil to a concentration of 2.0 mg/ml as described in several recent studies to be administered orally or subcutaneously in rodents [19]. Piceid and L-dopa were suspended in a 0.5% sodium carboxymethylcellulose (CMC-Na) solution.

#### Systemic rotenone model of PD and treatments

Male SD rats (weighing 220–240 g) were randomly allocated to six different groups (13–16 animals per group). The control group received a vehicle of sunflower oil 1.0 ml/kg/d subcutaneously (s. c.) and a vehicle of 0.5% CMC-Na 10 ml/kg/d orally for 5 weeks. PD neurodegeneration was induced by rotenone s. c. in a dose of 2.0 mg/kg/d in sunflower oil (1.0 ml/kg/d) s.c. plus 0.5% CMC-Na 10 ml/kg/d orally for 5 weeks. L-dopa and piceid treatments were performed orally in a dose of 10 mg/kg/d and 20, 40, 80 mg/kg/d in 0.5% CMC-Na 10 ml/kg/d, respectively, for the entire 5 weeks.

#### Motor behavioral tests

##### Catalepsy test

The development of parkinsonism was detected by the occurrence of tremors and the observation of bradykinesia and rigidity in rats were further quantified by a “Catalepsy test”. The first part was the grid test where each rat was hung by all four paws on a vertical grid (25.5 cm wide and 44 cm high with a space of 1 cm between each wire), and a stopwatch was started as soon as the rat held onto the grid. The time for the rats to move its paws or any sort of first movement was recorded as descent latency.

The second part was the bar test where the rats were placed with both forepaws on a bar which was 9 cm above and parallel from the base in a half rearing position and they were timed with a stopwatch. When the animals removed one paw from the bar the stopwatch was stopped and the latency noted. The maximum descent latency was fixed at 180 s for both tests. Both tests were repeated once a week to evaluate the disease progress, starting the first week.

##### Rotarod motor coordination test

The effect of rotenone on motor coordination and muscle performance was evaluated using a rotarod (DXP-2) test. The rotarod treadmill consists of a plastic rod, 9 cm in diameter and 36 cm long, with a non-slippery surface 20 cm above the base. This rod is divided into four equal sections by five discs (25 cm in diameter), which enables four rats to walk on the rod at the same time. In the present study, all rats were given two initial training trials of 300 s, approximately 10 min apart, to maintain posture on the rotarod (rotating at a constant 20 revolutions per minute). After the initial training trials, a baseline trial of 180 s was conducted. The time each animal remained on the rotarod was recorded by an observer blinded to the treatment groups; animals not falling off the rotarod were given a maximum score of 180 s. The test was repeated once a week to evaluate motor performance.

##### Preparation of brain homogenate from rotenone study

At the end of the 5 weeks, all rats were euthanized and brain tissues were collected: striatum, cortex and hippocampus of the two hemispheres were quickly excised and weighed. Tissues from half brains were placed in ice-cold saline (10% w/v) and homogenated using an electric glass homogenizer, followed by centrifugation at 10000 × g for 15 min, 4°C. Since this assay is not feasible in a denatured environment, no sodium dodecyl sulfate (SDS) was added to the lysate buffer. The resulting supernatant was used for the assays of ROS parameters. Tissues from the other half brains were used in the preparation of protein lysates for use in Western blot analysis.

##### Lipid peroxidation assay

Malondialdehyde (MDA), an end product of the polyunsaturated fatty acid oxygenation and a reliable and commonly used biomarker for assessing lipid peroxidation, was determined by thiobarbituric acid reactive substances (TBARS) according to a published method [50] (using a commercial kit (Cat#20091014, Nanjing Jiancheng Biotechnology Institute, China). In brief, 10% tissue homogenates or MDA standard were mixed with 100 μl of SDS. Then, 2.5 ml of a TBA/Buffer reagent mix was added and incubated in a boiling water bath for 80 min. The reaction mixture was centrifuged and its absorbance was measured at 532 nm. The MDA levels are expressed as MDA nmol/mg of proteins.

##### Superoxide dismutase (SOD) activity assay

The activity of SOD was determined based on the inhibitory effect of SOD on nitro blue tetrazolium (NBT) reduction by the superoxide anions generated by the photo-oxidation of hydroxylamine hydrochloride. The assessment was conducted by using the standard kit supplied by Nanjing Jiancheng Biotechnology Institute (Cat #20091013, China). Briefly, the assay system consisted of EDTA 0.1 mM, sodium carbonate 50 mM and 96 mM of NBT. In the cuvette, 2 ml of the above mixture, 0.05 ml of hydroxylamine and 0.05 ml of 1% brain homogenate were combined and the auto-oxidation of hydroxylamine was observed by measuring the absorbance at 550 nm. SOD activity was expressed as U/mg proteins.

##### Reduced glutathione assay

The amount of reduced glutathione (GSH) was determined by the amount of total non-protein sulfhydryl groups. In this method, GSH reacted with 5, 5′dithio-bis-(2-nitrobenzoic acid) (DTNB) to develop color absorbance that was measured at 420 nm. The assessment was conducted by using standard kits supplied by Nanjing Jiancheng Biotechnology Institute (Cat #20091125, China), following the manufacturer’s instructions. Briefly, 0.5 mL 10% tissue homogenate was immediately precipitated with 2 ml of 25% TCA and the precipitate was removed by centrifugation at 1500 × g for 10 min. 1 ml of the supernatant was added to 1 ml of DTNB. The absorbance of the yellow color complex was read at 420 nm. Content of GSH was expressed as mg GSH/mg proteins.

##### Adenosine triphosphate content assay

Adenosine triphosphate (ATP) levels in the brain tissues were determined spectrophotometrically using a kit (Nanjing Jiancheng Biotechnology Institute, Cat#20091126, China). The principle is based on the reaction between 3-phosphoglycerate and ATP catalyzed by phosphoglycerate kinase. The reaction was coupled with a dephosphorylation reaction using the enzyme glyceraldehyde phosphate dehydrogenase (GAPD) that involved the oxidation of NADH. Formation of NAD was then quantitated by measuring the decrease in absorbance at 340 nm, as a measure of the amount of ATP. The level of ATP was expressed as nmol ATP/mg proteins.

##### Western blot analysis of thioredoxin (Trx) expression

For The hemispheres collected at the end of the 5 week study (rotenone/piceid) were further dissected to separate striatum, cortex and hippocampus, samples were pooled together from 9 rats each group and 75 μg of protein was loaded for Western blot analysis using a rabbit polyclonal anti-Trx (1:1,000; Abcam, Hong Kong, Ltd.). Densitometric analysis was performed using the Quantity One 4.6.2 software.

##### Histological analysis of striatal tissues

At the end of the experiment (5 weeks), five rats from each group were perfused and processed for paraffin embedding. Mid-brain sections (coronal section of 8 ucrom thickness) were stained with Hematoxylin and Eosin (H& E) as well as with an anti-tyrosine hydroxylase/TH antibody (1:100; Sigma), followed by visualization with 3, 3′-diaminobenzidine (Sigma/Aldrich).

#### 6-OHDA induced PD model

Male SD rats (weighing 220–240 g) were randomly divided into control and model groups (n = 9-10 control and n > 60 model group). All rats were anesthetized by intraperitoneal injection of 10% chloral hydrate 3.5 ml/kg, and were stereotaxically fixed and injected with 6-OHDA into the right substantia nigra of brains (8 μg in 4 μl at 0.5 ul/min speed) [anterior fontanelle 4.4 mm, sagittal suture (right) 1.3 mm, 8.5 mm under the skull]. Seven days later, rats were intraperitoneally injected with 0.5 mg/kg apomorphine to induce rat rotating to the contralateral side and their rotary motion was recorded 30 min later. The rats that rotated greater than 7 times per minute passed this test and were continued to be tested on rotarod test once weekly until 28 days. On day 8 at the time of apomorphine injection, the 6-OHDA induced PD rats were further divided into 4 groups (n = 9–10 each group) and received daily oral treatment [piceid(50 mg/kg, l00 mg/kg, L-Dopa (25 mg/kg), L-Dopa (12.5 mg/kg) + Piceid (50 mg/kg)] starting a few hours after 6-OHDA injection. Their rotary motion was recorded on 7d, 14d, 21d and 28d.

#### MPTP-induced mouse model of PD and piceid treatment

Male C57BL/6 mice (>25 g) were randomly divided into three groups, namely the control group, model group and treatment group (piceid 100 mg/kg, piceid 200 mg/kg, L-Dopa 50 mg/kg, L-dopa 25 mg/kg + piceid 100 mg/kg). While the control group received saline by oral gavage, the model and treatment groups received ip injected MPTP at 20 mg/kg (0.1 ml/10 g) daily for 7 days. Daily compound treatments began on day 1 and continued for 14 days. Motor function was assessed on the 15^th^ day by their locomotor activity counts. In brief, homemade 30 cm × 30 cm × 15 cm plexiglass box which was carved out of the 6 cm × 6 cm grid at the bottom of box. Mice were tested in a quiet, low-light environment and were allowed to be adapted to the environment for 10 min. Their mobile grid number within 5 min was counted five times and the mean values were taken. Mice were also tested on rotarod test to determine motor coordination. They were placed on a rotating rod instrument (roller diameter of 6 cm, speed 13 r/min) one hour after of the last compound/drug administration and were automatically recorded (maximum detection time 180 s, than those who still denoted 180 s). Mice were euthanized immediately after the behavioral tests and the activity of SOD and the content of MDA of striatum were determined as described. For histology, coronal sections (6 μcrom) from paraffin-embedded mid-brain tissue were stained with H & E, mouse-anti-TH (Millopore, 1:200), rabbit anti-p-Akt (Cell Signaling) and rabbit anti-caspase −3 (Cell Signaling), followed by DAB or fluorescent secondary antibodies.

##### Statistical analysis

All data are presented as mean ± SEM. The data from the *in vivo* experiments (Figures 2, 3, 4 and 5) were analyzed using a one-way analysis of variance (one-way-ANOVA) followed by Newman-Keuls tests for post hoc comparisons between groups, with the exceptions of the data of grid test, bar test and rotarod test, which were analyzed by two-way repeated-measures ANOVA. All the statistical analyses were conducted using the Statistical Program of Social Sciences (SPSS) for windows, version 13.0 and values of *p* < 0.05 were considered to be significant.

## Electronic supplementary material

Additional file 1: **Online Methods. Figure S1.** Immuno double staining of TH and α-synuclein. **Figure S2.** Effect of Piceid treatment in MPTP-induced PD mice. **Figure S3.** Effect of Piceid treatment in rotenone-induced PD rats. **Figure S4.** Effect of Piceid treatment in 6-OHDA-induced PD rats. (PDF 345 KB)

## Abbreviations

PD: Parkinson’s disease
ROS: Reactive oxidative stress
L-dopa: L-levodopa
SNc: Substantia nigra
Trx: Thioredoxin; MDA
Rot: Rotenone
Rsv: Resveratrol’
Pic: Piceid
MPTP: Malondialdehyde.
