# Supplementary material for: Anti-oxidant polydatin (piceid) protects against substantia nigral motor degeneration in multiple rodent models of Parkinson’s disease
Source: Mol Neurodegener. 2015 Mar 2;10:4. doi: 10.1186/1750-1326-10-4 (PMC4506434; doi:10.1186/1750-1326-10-4)
Supplement: Supplementary file 1 — Additional file 1: Online Methods. Figure S1. Immuno double staining of TH and α-synuclein. Figure S2. Effect of Piceid treatment in MPTP-induced PD mice. Figure S3. Effect of Piceid treatment in rotenone-induced PD rats. Figure S4. Effect of Piceid treatment in 6-OHDA-induced PD rats. (PDF 345 KB) [file 13024_2013_580_MOESM1_ESM.pdf]

Additional file 1

Online Methods

Quantitative Histology

Fixed brain tissues were cryoprotected and sectioned at 40 ucrom coronally and subjected to immunofluorescent staining using antibodies against TH (Immunostar #22941) and a-synuclein (C20, sc-7011-R; Santa Cruz), both at 1:100 dilution. Images were captured from Confocal microscope. Quantification was conducted using stereological principles (5 animals/group).

Statistical analyses were performed using unpaired two-tailed or one-tailed Student's *t*-test. Data were reported as mean ±S.E.M. Statistical significance was accepted at $P<0.05$.

Figure Legends

Figure S1. Immuno double staining of TH and α-synuclein. Staining was performed and optimized on mid brain sections from α-synuclein transgenic mice generated previously (Lin 2012). Colocalization of these two antigens was found in dopaminergic neurons, in particular in older mice when α-synuclein pathology prevailed in the remaining TH neurons.

Supplementary Figure 1

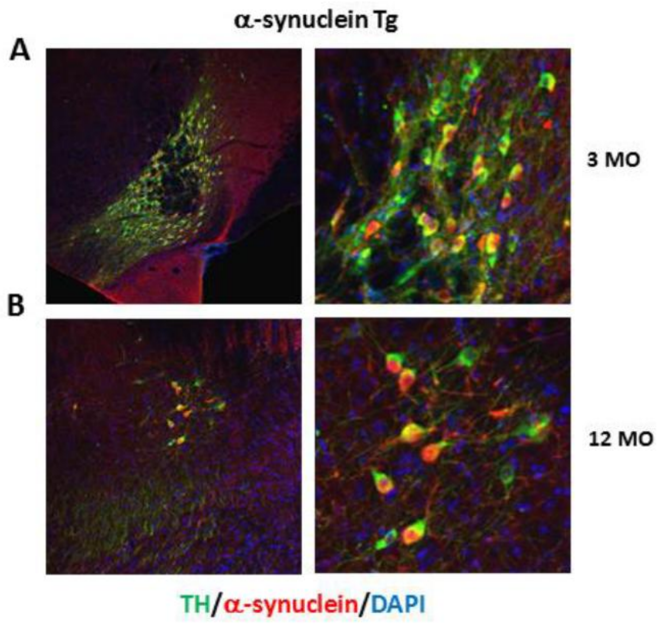

Figure S2. Effect of Piceid treatment in MPTP-induced PD mice. Daily ip injection of MPTP (20 mg/kg) for a continuous 7 days caused massive loss of TH neurons as well as weak α-synuclein staining in the SNc of male C57Bl/6 mice which were found to be largely prevented by daily piceid treatment (100 mg/kg, gavage). ** P< 0.01.

Supplementary Figure 2

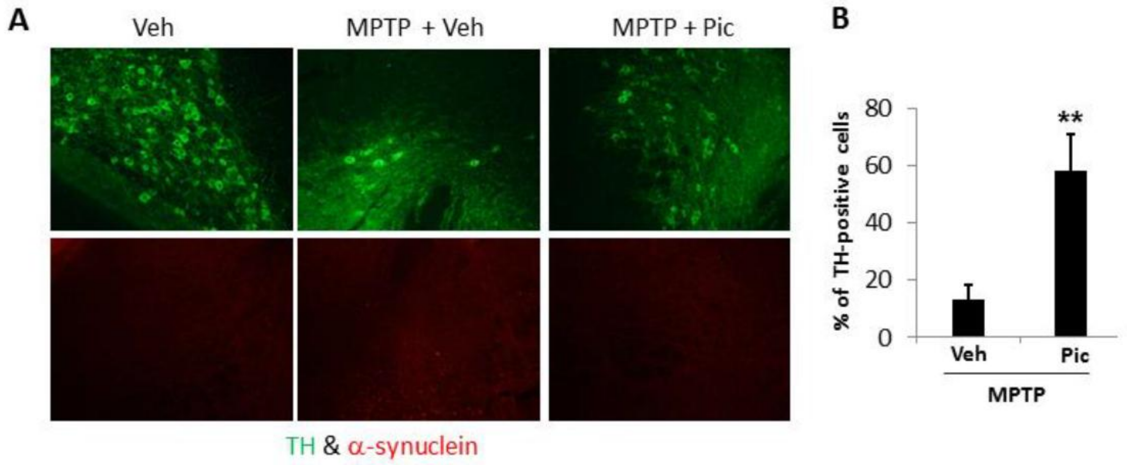

Figure S3. Effect of Piceid treatment in rotenone-induced PD rats. No α-synuclein pathology was found in SD rats after 5-week rotenone (2.0 mg/kg daily s. c.) induction. Piceid largely rescued rats from dopaminergic neurodegeneration.

Supplementary Figure 3

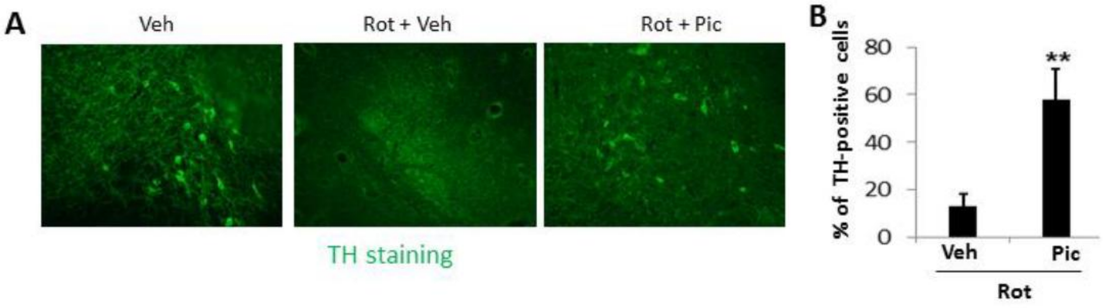

Figure S4. Effect of Piceid treatment in 6-OHDA-induced PD rats. Daily piceid treatment starting 2 hours after 6-OHDA injection for 7 days only partially protected dopaminergic neurons in VTA region but not in the SNc region.

Supplementary Figure 4

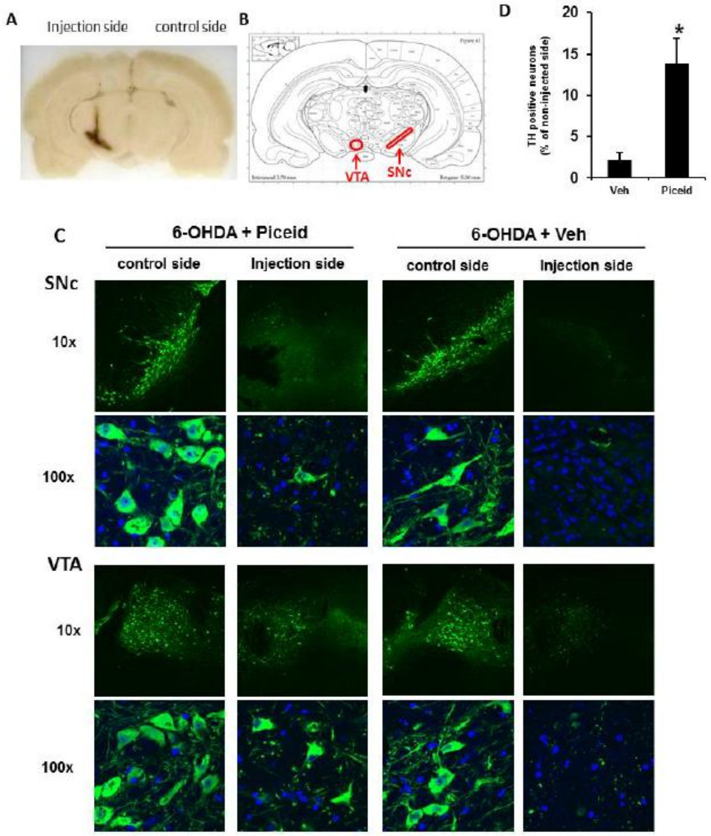

Reference

Lin X, Parisiadou L, Sgobio C, Liu G, Yu J, Sun L, Shim H, Gu XL, Luo J, Long CX, Ding J, Mateo Y, Sullivan PH, Wu LG, Goldstein DS, Lovinger D, Cai H: **Conditional expression of Parkinson's disease-related mutant α-synuclein in the midbrain dopaminergic neurons causes progressive neurodegeneration and degradation of transcription factor nuclear receptor related 1,** *J Neurosci* 2012, 32:9248-64.
